# Dyadic associations of physical activity intensity with fertility-related quality of life in infertile couples: an APIM analysis

**DOI:** 10.3389/fpubh.2026.1923237

**Published:** 2026-08-24

**Authors:** Fei Jiang, Zhengmei Liao, Yanan Chen, Qiufeng Gu, Guiying Luo, Qianhua Xu, Jieyu Wang, Danni Wang

**Affiliations:** 1 Department of Nutrition, School of Public Health, Anhui Medical University, Hefei, Anhui, China; 2 Department of Health Promotion and Behavioral Sciences, School of Public Health, Anhui Medical University, Hefei, Anhui, China; 3 Reproductive Medicine Center, Department of Obstetrics and Gynecology, The First Affiliated Hospital of Anhui Medical University, Hefei, Anhui, China

**Keywords:** actor-partner interdependence model, assisted reproductive technology, infertility, physical activity, quality of life

## Abstract

**Objective:**

Infertility is a condition that intrinsically affects both members of a couple. However, the dyadic associations between physical activity intensity and fertility-related quality of life (FertiQoL) remains insufficiently explored. This study aimed to examine dyadic associations between physical activity intensity and FertiQoL among infertile couples undergoing assisted reproductive technology (ART) treatment.

**Methods:**

This cross-sectional study included 364 infertile couples receiving ovulation induction treatment at a reproductive medicine center. Physical activity levels and FertiQoL were assessed using validated questionnaires. Multivariable linear regression was used to examine overall associations between physical activity and FertiQoL domains. The Actor–Partner Interdependence Model (APIM) was applied to estimate actor associations (individual physical activity with own FertiQoL) and partner associations (individual physical activity with spouse’s FertiQoL).

**Results:**

Women reported significantly lower physical activity levels and FertiQoL scores compared to men (all *p* < 0.05). Overall, higher total physical activity (TPA) and most intensity levels were positively associated with multiple FertiQoL domains. The adjusted APIM analysis demonstrated a significant actor association among husbands, whereby higher light physical activity (LPA) was associated with better own FertiQoL (*β* = 0.205, 95% CI: 0.076 to 0.335, *p* = 0.002). Notably, distinct intensity-dependent partner effects were observed: wives’ moderate physical activity (MPA) was positively associated with husbands’ FertiQoL (*β* = 0.150, 95% CI: 0.024 to 0.277, *p* = 0.021), whereas wives’ vigorous physical activity (VPA) was negatively associated with husbands’ FertiQoL (*β* = −0.184, 95% CI: −0.307 to −0.061, *p* = 0.004).

**Conclusion:**

Physical activity is significantly associated with quality of life among infertile couples in an intensity-dependent manner. More importantly, physical activity demonstrates dyadic interdependence, with wives’ physical activity exerting intensity-specific effects on husbands’ FertiQoL. These findings highlight the importance of considering couple-level lifestyle behaviors in infertility care and suggest that dyadic lifestyle approaches warrant further evaluation as potential supportive strategies during ART treatment.

## 1 Introduction

The incidence of infertility is increasing annually, with a notable shift toward younger populations, posing a profound threat to global public health (1). Current estimates indicate that the prevalence of infertility ranges from 3.5 to 16.7% in developed countries and from 6.9 to 9.3% in developing regions (2). Globally, this affects approximately 48.5 to 72.4 million couples (3), representing nearly one in seven couples of reproductive age (4). In this context, infertile couples often experience a diminished quality of life (QoL), particularly women (5, 6). As a multidimensional construct, QoL encompasses an individual’s perceived functioning across psychological, physiological, social, and environmental domains (7). Although not life-threatening, infertility contributes to significant psychological burdens and chronic stress secondary to impaired reproductive function, undermining overall health status to some extent (8, 9). In response to these challenges, assisted reproductive technology (ART) has integrated clinical diagnostics, laboratory procedures, nursing, and counseling (10), leading to over 8 million births globally by 2020 (11). However, current services frequently struggle with limited treatment modalities and suboptimal patient experiences, often neglecting the multifaceted physical, psychological, and social demands of infertile couples (12, 13). Notably, fertility-related quality of life (FertiQoL), a measure of quality of life in people experiencing fertility problems, has been identified as a key predictor of clinical outcomes, including ongoing pregnancy and live birth (14, 15). Therefore, enhancing FertiQoL is essential for improving reproductive health issue among those struggling with infertility.

Physical activity has emerged as a cornerstone intervention in human health research (16). Nevertheless, insufficient physical activity remains a global concern, affecting approximately 27.5% of the population (17). A growing body of evidence suggests that physical activity is strongly associated with a broad spectrum of health outcomes, including obesity (18), metabolic syndrome (19), cardiovascular diseases (20), chronic non-communicable diseases (21), musculoskeletal disorders (22), cancers (23), quality of life (24), cognitive and mental disorders (20, 25). In reproductive health, physical activity has been shown to reduce infertility risk and enhancing clinical pregnancy rates (26, 27). Consequently, it serves as an economical and accessible complementary therapy in reproductive care (28). A growing documentation further supports its positive individual-level benefits on QoL. For instance, recreational physical activity is positively associated with both general and FertiQoL in women undergoing fertility treatment (29), whereas physical inactivity was significantly related to poorer health-related quality of life in women with polycystic ovary syndrome (30). However, the majority of prior studies have treated husbands and wives as isolated entities by focusing exclusively on individual outcomes, particularly among female participants, thereby leaving couple-level dynamics and intensity-specific patterns underexplored (31).

Theoretically, infertility is fundamentally a shared stressor that profoundly impacts the QoL of both partners as an interdependent unit (32). According to the Actor–Partner Interdependence Model (APIM) proposed by Kenny et al. (33), dyadic individuals in intimate relationships engage in strong interpersonal interactions. An individual’s emotions, cognitions, or behaviors can readily transfer between partners and ultimately influence each other’s health outcomes. Within this framework, the effect of an individual’s state on their own outcome is defined as the actor effect, whereas the effect of an individual’s state on their partner’s outcome is defined as the partner effect. In line with this theoretical perspective, accumulating empirical evidence confirms that health outcomes, psychological states, and health behaviors are deeply interdependent between spouses. Specifically, infertile couples’ QoL is shaped by both partners’ stress and dyadic coping, where wives’ coping directly impacts husbands’ QoL, and husbands’ stress and coping similarly affect wives (32). Similarly, dyadic coping exerts significant actor and partner effects on anxiety and depression in infertile couples (34). Gender-specific patterns further show that wives’ depressive symptoms relate solely to their own behavioral patterns, whereas husbands are influenced by both spouses’ behaviors, highlighting the dyadic impact of health behaviors (35). In terms of physical activity, research demonstrates that husbands’ physical activity and depressive symptoms predict wives’ physical activity and depressive symptoms, respectively (36). Furthermore, among older couples with chronic conditions, low physical activity in patients is associated with increased depressive symptoms in their spouses, highlighting the importance of joint physical activity for partner well-being (37). At the individual level, higher physical activity intensity directly improves one’s own FertiQoL by alleviating stress pathways, mitigating anxiety, and reducing depressive symptoms. At the partner level, given the strong mood and behavioral synchronization within couples, the physical well-being and stress reduction derived from physical activity induce a positive emotional spillover, thereby potentially enhancing the spouse’s QoL. Based on this, the present study aimed to examine the associations between physical activity intensity and FertiQoL in an infertile population. Furthermore, applying the APIM, we sought to investigate the relationships between individuals’ physical activity patterns and their partners’ FertiQoL by evaluating both actor and partner patterns within the couple unit.

## 2 Methods

### 2.1 Participants

**Figure 1:**
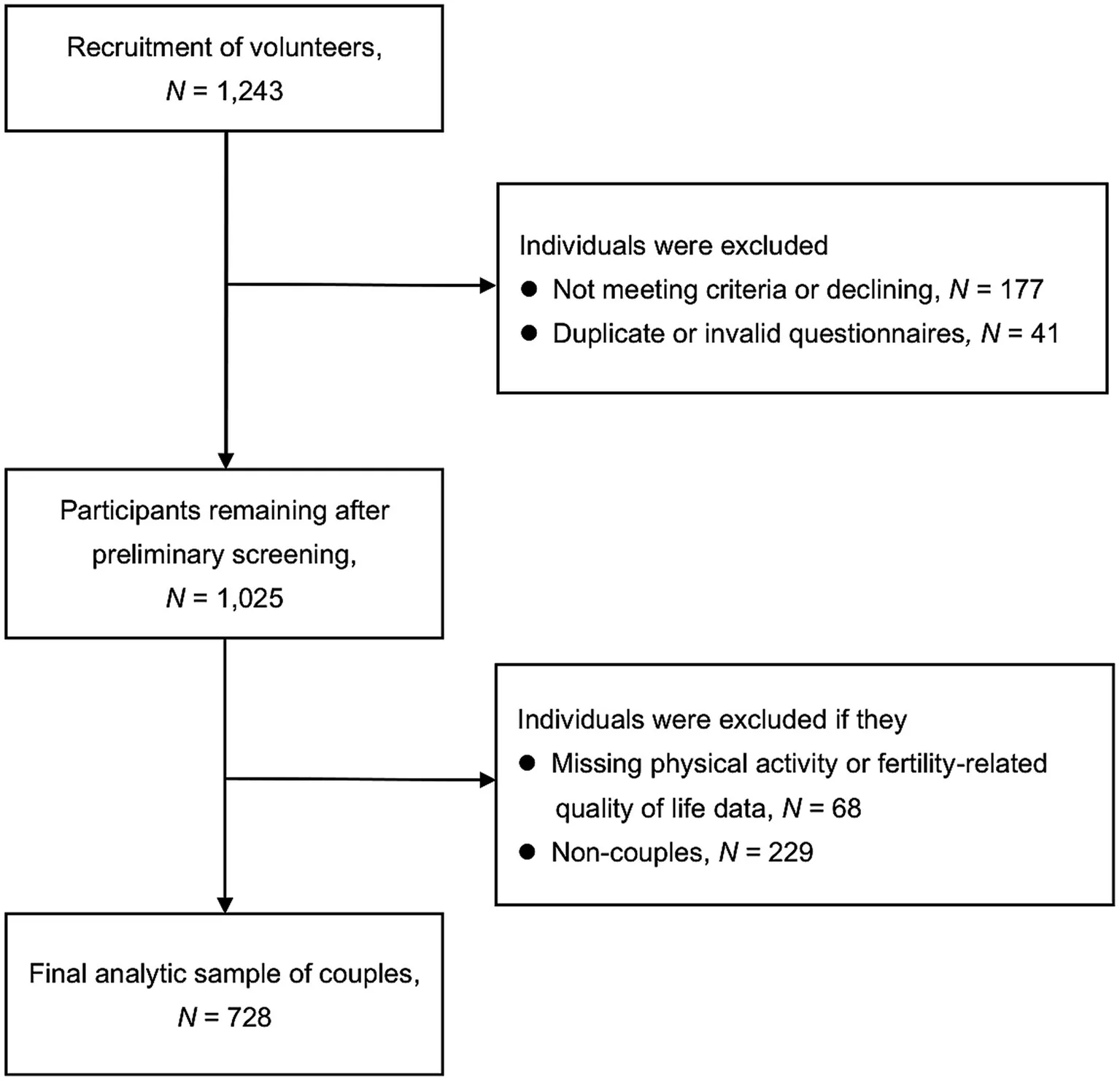
Flowchart of the participant selection process.

Data for this cross-sectional study were collected from infertile couples seeking ART treatments at the Center for Reproductive Medicine of the First Affiliated Hospital of Anhui Medical University between July and October 2022. Inclusion criteria required that participants (1) met the medical definition of infertility, defined as the failure to achieve clinical pregnancy after 12 months or more of regular unprotected sexual intercourse (38); (2) had no history of psychiatric disorders; (3) were actively seeking fertility treatment; (4) provided written informed consent; and (5) were capable of completing the survey. Conversely, individuals were excluded if they presented with medical comorbidities such as hypertension, diabetes, or kidney disease, had missing data on physical activity or QoL, or if their partners did not participate in the study. Eligible participants were recruited during their clinical visits and were specifically in the ovulation induction phase of their treatment. Under the supervision of a team of two or three trained investigators, participants completed an electronic questionnaire and underwent physical examinations. The final analytic sample consisted of 728 individuals from 364 infertile couples (Figure 1). This study followed the guidelines of the Declaration of Helsinki and was approved by the Ethics Committee of Anhui Medical University (No. 20200961). Written informed consent was obtained from all participants prior to enrollment.

### 2.2 Quality of life assessment

The QoL of the study participants was assessed using the simplified Chinese version of the FertiQoL questionnaire, developed by the European Society of Human Reproduction and Embryology (ESHRE) and the American Society for Reproductive Medicine (ASRM) (39). The FertiQoL consists of 34 items divided into two modules: the Core FertiQoL (24 items, encompassing Emotional, Mind–Body, Relational, and Social domains) and the Treatment FertiQoL (10 items, encompassing Environment and Tolerability domains). The simplified Chinese version of the FertiQoL questionnaire has demonstrated good reliability and validity (40, 41). In our survey sample, the overall instrument exhibited excellent internal consistency (Cronbach’s *α* = 0.948), with subscale Cronbach’s α values of 0.825 for Emotional, 0.913 for Mind–Body, 0.620 for Relational, 0.723 for Social, 0.778 for Environment, and 0.858 for Tolerability domains. Regarding item scoring and transformation, all items were measured on a 5-point Likert scale ranging from 0 to 4. Prior to score calculation, negatively worded items were reverse-scored (4 to 0) so that higher scores consistently represented better quality of life. Raw subscale and total scores were computed by summing the relevant item scores. To standardize the results, raw scores were linearly converted to a 0–100 scale using the formula: (sum of raw item scores) × 25 / *k*, where *k* represents the number of items in the respective domain or module (e.g., *k* = 24 for Core FertiQoL, *k* = 10 for Treatment FertiQoL, and *k* = 34 for Total FertiQoL).

### 2.3 Physical activity assessment

Physical activity levels were assessed using a validated, structured physical activity questionnaire (PAQ) with a 1-year recall period. Participants reported their average weekly duration for specific activities over the past year using a 10-level scale ranging from 0 to over 300 minutes. Evaluated activities covered multiple domains, including walking (classified into 5 levels ranging from mobility-impaired to very brisk), running (including outdoor running and treadmill exercise), bicycling, racquet and team sports (e.g., basketball, soccer, tennis, and table tennis), swimming, aerobic exercises, high-intensity interval training (HIIT), traditional and mind–body exercises (e.g., Tai Chi, yoga, and square dancing), domestic activities (e.g., cooking and cleaning), heavy carrying, and resistance training (e.g., dumbbells, squats, and weighted leg exercises), as well as stationary daily activities (e.g., desk work and screen-based behaviors). Metabolic equivalent of task (MET) values for each activity were assigned according to the 2011 Compendium of Physical Activities (42), as detailed in Supplementary Table 1.

Physical activity volume was expressed as MET-hours per week (MET-h/week). The weekly volume of physical activity for each activity was calculated by multiplying its assigned MET value by the reported weekly duration (in hours). Based on MET thresholds, physical activity was assessed across light physical activity (LPA, < 3.0 METs), moderate physical activity (MPA, 3.0–5.9 METs), vigorous physical activity (VPA, ≥ 6.0 METs), and moderate-to-vigorous physical activity (MVPA, ≥ 3.0 METs), along with total physical activity (TPA). The reliability and validity of PAQ have been established previously against 24-h physical activity recalls and 7-day physical activity diaries (43, 44). This instrument is widely used in Anhui Province, China, and demonstrated good internal consistency in our sample (Cronbach’s *α* = 0.816).

### 2.4 Covariates

Potential covariates were selected from self-reported questionnaires and physical measurements based on previous literature. Demographic and socioeconomic factors included age, gender (male or female), education level (middle school or below, high/vocational school, or college degree or above), and annual income (< 30,000, 30,000-60,000, or > 60,000 CNY). Health-related and lifestyle variables encompassed body mass index (BMI) (< 18.5, 18.5–23.9, 24.0–27.9, ≥ 28 kg/m^2^), smoking status (yes or no), alcohol consumption (yes or no) and sleep quality (good or poor). Depressive symptoms were assessed using the nine-item Patient Health Questionnaire (PHQ-9), with a total score of ≥ 5 indicating the presence of at least mild depression (yes or no). Clinical and fertility-related characteristics included marital status (first marriage or remarriage), presence of living children (yes or no), family support for ART (supportive or opposing), history of overnight injections (yes or no), cause of infertility (male factor, female factor, both, or unexplained), duration of infertility (≤ 1, 1–2, or > 2 years), and duration of infertility treatment (≤ 1, 1–2, or > 2 years).

### 2.5 Statistical analysis

Continuous variables were expressed as mean ± standard deviation (SD) for normally distributed data, and compared using the *t*-test or one-way analysis of variance (ANOVA). Non-normally distributed continuous variables were presented as median and interquartile range (IQR), with differences evaluated using the Wilcoxon rank-sum test or Kruskal-Wallis test. Categorical variables were described as frequencies and percentages (%), and compared using the chi-square test or Fisher’s exact test. The association between physical activity and quality of life was analyzed using multivariable linear regression models, adjusting for potential confounders including age, gender, BMI, education, annual income, marital status, smoking status, alcohol consumption, sleep quality, depressive symptoms, presence of living children, family support for ART, history of overnight injections, cause of infertility, and the duration of both infertility and its treatment. Furthermore, structural equation modeling (SEM) based on the APIM was performed to examine the actor and partner associations of physical activity intensities on FertiQoL within infertile dyads. Specifically, physical activity intensities including LPA, MPA, and VPA were specified as exogenous observed predictor variables for both husbands and wives. FertiQoL was modeled as an endogenous latent outcome variable, measured by its corresponding observed subscale indicators. Based on preliminary multiple linear regression results, and to maintain model parsimony while avoiding overparameterization in the SEM, key sociodemographic variables including age, BMI, education, annual income, and marital status were incorporated as covariates. In this model, dyads were specified as distinguishable by gender (husband vs. wife) and uniquely linked using paired couple identification codes. Missing data (n = 68) were handled using listwise deletion for a complete-case analysis, given the low missingness rate (<10%) and the requirement for intact paired data in dyadic SEM. All statistical analyses were conducted using SPSS version 23.0 and R version 4.3.1. All tests were two-sided, with statistical significance defined as *p* < 0.05.

## 3 Results

### 3.1 Basic characteristics

The baseline characteristics of the 728 enrolled participants are summarized in Table 1. Overall, the mean age of the study population was 30.87 ± 4.28 years. Notably, females were significantly younger than males (30.46 ± 4.00 vs. 31.28 ± 4.52, *p* = 0.010). Significant gender-specific differences were observed in BMI, annual income, marital status, smoking status, alcohol consumption, sleep quality, depressive symptoms, and primary cause of infertility (all *p* < 0.05).

**Table 1:** Baseline characteristics of the study participants by gender (*N* = 364 dyads).

Characteristic	Overall (*n* = 728)	Female (*n* = 364)	Male (*n* = 364)	*p*
Age (years), mean ± SD	30.87(4.28)	30.46(4.00)	31.28 (4.52)	0.010
BMI (kg/m^2^), mean ± SD	23.87 (5.09)	22.34 (4.4)	25.44 (4.17)	< 0.001
Education, *n* (%)				0.655
Middle school or below	201 (27.6)	103 (28.3)	98 (26.9)	
High/vocational school	272 (37.4)	130 (35.7)	142 (39.0)	
College degree or above	255 (35.0)	131 (36.0)	124 (34.1)	
Annual income (CNY), *n* (%)				< 0.001
< 30,000	252 (34.6)	162 (44.5)	90 (24.7)	
30,000-60,000	169 (23.2)	98 (26.9)	71 (19.5)	
> 60,000	307 (42.2)	104 (28.6)	203 (55.8)	
Marital status, *n* (%)				0.035
First marriage	665 (91.3)	324 (89.0)	341 (93.7)	
Remarriage	63 (8.7)	40 (11.0)	23 (6.3)	
Smoking status, yes, *n* (%)	148 (20.3)	3 (0.8)	145 (39.8)	< 0.001
Alcohol consumption, yes, *n* (%)	53 (7.3)	4 (1.1)	49 (13.5)	< 0.001
Sleep quality, good, *n* (%)	650 (89.3)	312 (85.7)	338 (92.9)	0.003
Depressive symptoms, yes, *n* (%)	368 (50.5)	165 (45.3)	203 (55.8)	0.005
Presence of living children, yes, *n* (%)	91 (12.5)	51 (14.0)	40 (11.0)	0.262
Family support for ART supportive, *n* (%)	663 (91.1)	326 (89.6)	337 (92.6)	0.194
History of overnight injections, yes, *n* (%)	634 (87.1)	322 (88.5)	312 (85.7)	0.320
Duration of infertility (years), *n* (%)				0.893
≤ 1	267 (36.7)	132 (36.3)	135 (37.1)	
1–2	225 (30.9)	111 (30.5)	114 (31.3)	
> 2	236 (32.4)	121 (33.2)	115 (31.6)	
Duration of infertility treatment (years), *n* (%)				0.823
≤ 1	130 (17.9)	55 (15.1)	75 (20.6)	
1–2	247 (33.9)	142 (39.0)	105 (28.8)	
> 2	187 (25.7)	92 (25.3)	95 (26.1)	
Cause of infertility, *n* (%)				0.020
Female factor	247 (33.9)	142 (39.0)	105 (28.8)	
Male factor	130 (17.9)	55 (15.1)	75 (20.6)	
Both	187 (25.7)	92 (25.3)	95 (26.1)	
Unexplained	164 (22.5)	75 (20.6)	89 (24.5)	

Data are presented as mean ± SD for continuous variables and *n* (%) for categorical variables. *p* < 0.05 indicates statistical significance. SD, standard deviation; BMI, body mass index; ART, assisted reproductive technology.

### 3.2 Distribution and correlations of physical activity and FertiQoL

As summarized in Table 2, significant gender differences were observed in physical activity intensities and FertiQoL. Specifically, females reported significantly lower levels of MPA, VPA, MVPA, and TPA than their male partners (all *p* < 0.001), whereas LPA levels did not differ significantly between sexes (*p* = 0.896). Regarding FertiQoL scores, females exhibited significantly lower scores in the emotional, mind–body, and tolerability domains, as well as lower Core and Total FertiQoL scores compared with males (all *p* < 0.01). Conversely, females (62.24 ± 14.11) scored significantly higher in the environment domain than males (61.46 ± 14.54, *p* = 0.045). No significant differences were found in the relational, social, or treatment domains (all *p* > 0.05). Furthermore, significant positive correlations were observed between partners for the same intensities of physical activity, with Spearman’s correlation coefficients (*r*_s_) ranging from 0.124 to 0.362 (all *p* < 0.05; Supplementary Table 2). Similarly, significant correlations were found between partners’ FertiQoL scores across identical dimensions, with Pearson’s correlation coefficients (*r*) ranging from 0.166 to 0.351 (all *p* < 0.05; Supplementary Table 3).

**Table 2:** Differences in physical activity intensities and FertiQoL scores between female and male partners (*N* = 364 dyads).

Variables	Female partner (n = 364)	Male partner (n = 364)	*Z/t*	*p*
Physical activity (MET-h/week)^a^				
LPA	10.95 (5.97, 20.55)	12.85 (5.43, 20.57)	−0.131	0.896
MPA	10.00 (5.25, 19.99)	14.15 (7.00, 24.13)	−5.549	<0.001
VPA	2.63 (0.00, 8.84)	7.75 (2.00, 20.22)	−6.195	<0.001
MVPA	14.50 (7.00, 29.81)	24.36 (12.51, 40.09)	−6.810	<0.001
TPA	28.15 (17.41, 47.39)	39.30 (23.92, 62.98)	−6.436	<0.001
FertiQoL scores^b^				
Emotional	62.13 ± 18.68	68.58 ± 19.83	5.040	<0.001
Mind–body	63.77 ± 21.70	71.83 ± 21.14	5.872	<0.001
Relational	62.72 ± 13.94	62.39 ± 12.55	−0.397	0.691
Social	68.80 ± 17.61	70.50 ± 17.73	1.612	0.108
Environment	62.24 ± 14.11	61.46 ± 14.54	−2.009	0.045
Tolerability	61.40 ± 19.90	65.13 ± 20.53	2.722	0.007
Core FertiQoL	64.29 ± 15.10	68.32 ± 15.41	4.334	<0.001
Treatment FertiQoL	62.45 ± 13.43	62.93 ± 13.56	0.573	0.567
Total FertiQoL	63.75 ± 13.68	66.74 ± 13.67	3.612	<0.001

^a^Continuous non-normally distributed data are presented as Median (IQR) and compared using the Wilcoxon signed-rank test (Z). ^b^Continuous normally distributed data are presented as Mean ± SD and compared using paired t-tests (t). *p* < 0.05 indicates statistical significance.

FertiQoL, fertility-related quality of life; LPA, light physical activity; MPA, moderate physical activity; VPA, vigorous physical activity; MVPA, moderate-to-vigorous physical activity; TPA, total physical activity.

### 3.3 Associations between physical activity and FertiQoL

**Figure 2:**
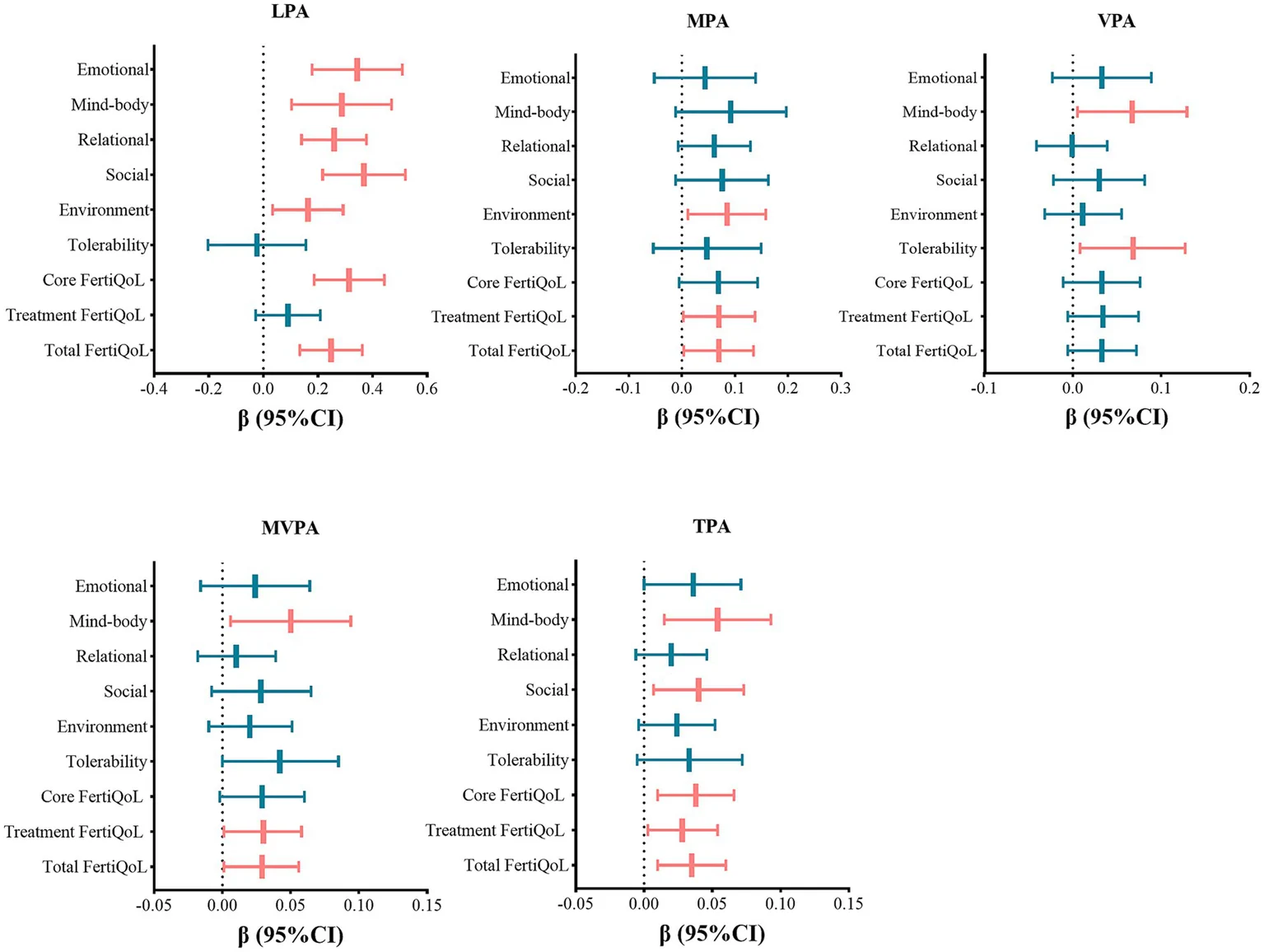
Forest plots showing the associations between physical activity intensities and FertiQoL scores. The model adjusted for age, gender, BMI, education, annual income, marital status, smoking status, alcohol consumption, sleep quality, depressive symptoms, number of living children, family members’ views on ART, timing of first trigger injection, cause of infertility, duration of infertility, and duration of infertility treatment. Abbreviations: FertiQoL, fertility-related quality of life; LPA, light physical activity; MPA, moderate physical activity; VPA, vigorous physical activity; MVPA, moderate-to-vigorous physical activity; TPA, total physical activity.

The multivariate linear regression results, adjusted for potential covariates, are summarized in Table 3 and Figure 2. Overall, TPA was positively associated with multiple FertiQoL domains: each 1 MET-h/week increase in TPA was linked to higher scores in the mind–body (*β* = 0.054, 95% CI: 0.015 to 0.093), social (*β* = 0.040, 95% CI: 0.007 to 0.073), core (*β* = 0.038, 95% CI: 0.010 to 0.066), and treatment FertiQoL (*β* = 0.028, 95% CI: 0.003 to 0.054) domains, as well as the total FertiQoL score (*β* = 0.035, 95% CI: 0.010 to 0.060), all with *p* < 0.05. Regarding specific physical activity intensities, LPA exhibited the most extensive associations, with each 1 MET-h/week increment significantly improving emotional (*β* = 0.344, 95% CI: 0.178 to 0.509), mind–body (*β* = 0.287, 95% CI: 0.103 to 0.470), relational (*β* = 0.259, 95% CI: 0.140 to 0.377), social (*β* = 0.368, 95% CI: 0.217 to 0.520), environment (*β* = 0.163, 95% CI: 0.034 to 0.292), core (*β* = 0.314, 95% CI: 0.186 to 0.443), and total FertiQoL scores (*β* = 0.248, 95% CI: 0.133 to 0.363), all with *p* < 0.05. In contrast, MPA contributed to higher scores in the environment, treatment, and total domains (*β* = 0.085, 95% CI: 0.011 to 0.158; *β* = 0.070, 95% CI: 0.003 to 0.138; and *β* = 0.070, 95% CI: 0.004 to 0.135, respectively, all *p* < 0.05). While VPA specifically enhanced mind–body and tolerability scores (*β* = 0.067, 95% CI: 0.005 to 0.129; and *β* = 0.068, 95% CI: 0.008 to 0.127, respectively, all *p* < 0.05). Finally, increased MVPA was associated with higher mind–body, treatment, and total FertiQoL scores (*β* = 0.050, 95% CI: 0.006 to 0.094; *β* = 0.030, 95% CI: 0.001 to 0.058; and *β* = 0.029, 95% CI: 0.001 to 0.056, respectively, all *p* < 0.05).

**Table 3:** Multivariate linear regression analysis of the associations between physical activity intensities and FertiQoL scores (*N* = 364 dyads).

FertiQoL	LPA *β* (95% CI)	MPA *β* (95% CI)	VPA *β* (95% CI)	MVPA *β* (95% CI)	TPA *β* (95% CI)
Emotional	0.344 (0.178, 0.509) ^* *^	0.044 (−0.052, 0.139)	0.033 (−0.023, 0.089)	0.024 (−0.016, 0.064)	0.036 (0.000, 0.071)
Mind–body	0.287 (0.103, 0.470) ^* *^	0.092 (−0.012, 0.197)	0.067 (0.005, 0.129) ^*^	0.050 (0.006, 0.094) ^*^	0.054 (0.015, 0.093) ^* *^
Relational	0.259 (0.140, 0.377) ^* *^	0.061 (−0.007, 0.129)	−0.001 (−0.041, 0.039)	0.010 (−0.018, 0.039)	0.020 (−0.006, 0.046)
Social	0.368 (0.217, 0.520) ^* *^	0.076 (−0.012, 0.163)	0.030 (−0.022, 0.081)	0.028 (−0.008, 0.065)	0.040 (0.007, 0.073) ^*^
Environment	0.163 (0.034, 0.292) ^*^	0.085 (0.011,0.158) ^*^	0.011 (−0.032, 0.055)	0.020 (−0.010, 0.051)	0.024 (−0.004, 0.052)
Tolerability	−0.023 (−0.202, 0.156)	0.047 (−0.054, 0.149)	0.068 (0.008, 0.127) ^*^	0.042 (0.000, 0.085)	0.033 (−0.005, 0.072)
Core FertiQoL	0.314 (0.186, 0.443) ^* *^	0.069 (−0.005, 0.143)	0.033 (−0.011, 0.076)	0.029 (−0.002, 0.060)	0.038 (0.010, 0.066) ^* *^
Treatment FertiQoL	0.090 (−0.028, 0.209)	0.070 (0.003, 0.138) ^*^	0.034 (−0.006, 0.074)	0.030 (0.001, 0.058) ^*^	0.028 (0.003, 0.054) ^*^
Total FertiQoL	0.248 (0.133, 0.363) ^* *^	0.070 (0.004, 0.135) ^*^	0.033 (−0.006, 0.072)	0.029 (0.001, 0.056) ^*^	0.035 (0.010, 0.060) ^* *^

Adjusted for age, gender, BMI, education, annual income, marital status, smoking status, alcohol consumption, sleep quality, depressive symptoms, number of living children, family members’ views on ART, timing of first trigger injection, cause of infertility, duration of infertility, and duration of infertility treatment. β indicates the unstandardized regression coefficient. *p* < 0.05 and *p* < 0.01 are indicated by * and **, respectively. FertiQoL, fertility-related quality of life; LPA, light physical activity; MPA, moderate physical activity; VPA, vigorous physical activity; MVPA, moderate-to-vigorous physical activity; TPA, total physical activity.

### 3.4 Dyadic analysis based on APIM

**Figure 3:**
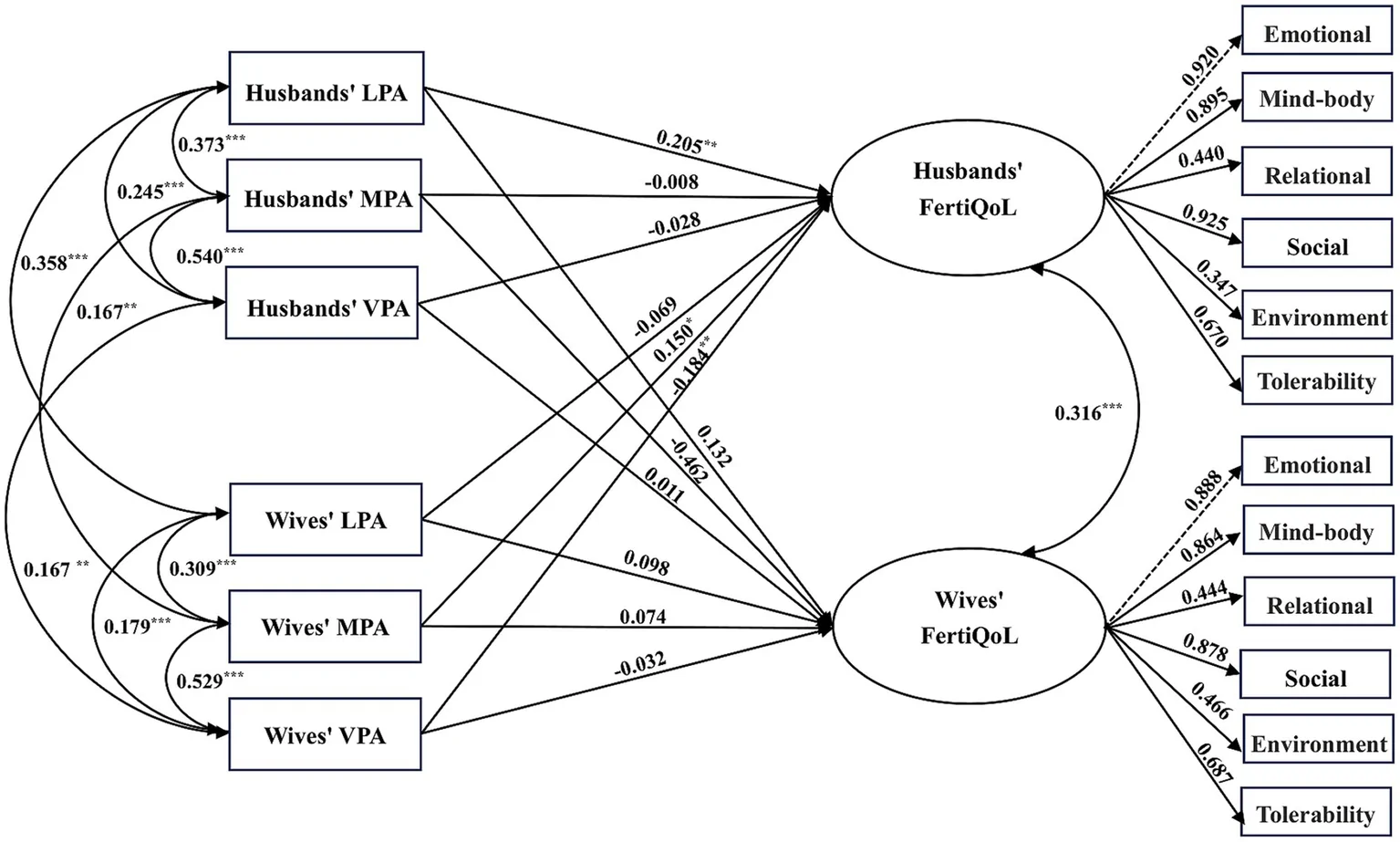
Structural equation model of the association between physical activity intensities and FertiQoL based on the APIM framework (*N* = 364 dyads). Single-headed arrows represent standardized path coefficients (*β*), and double-headed curved arrows represent standardized correlation coefficients (*r*). Rectangles represent observed exogenous manifest variables (LPA, MPA, and VPA), whereas large ellipses represent endogenous latent constructs (Husbands’ FertiQoL, Wives’ FertiQoL), each measured by six observed subscale indicators (Emotional, Mind–body, Relational, Social, Environmental, and Tolerability). Covariates (age, BMI, educational level, annual household income, and marital status) were adjusted for in the model but omitted from the figure for visual simplicity. *p* < 0.05, *p* < 0.01, and *p* < 0.001 are indicated by *, **, and ***, respectively. Abbreviations: FertiQoL, fertility-related quality of life; LPA, light physical activity; MPA, moderate physical activity; VPA, vigorous physical activity.

As shown in Table 4 and Figure 3, the unadjusted model (Model 1) revealed similar actor-partner patterns, where husbands’ LPA and wives’ MPA showed positive links with FertiQoL, while wives’ VPA showed an inverse relationship. These associations remained robust after adjusting for covariates (Model 2). Specifically, path coefficients for Model 2 indicated a significant actor link for husbands, with higher LPA positively connected with their own FertiQoL (*β* = 0.205, 95% CI: 0.076 to 0.335, *p* = 0.002). Regarding partner patterns, wives’ MPA remained positively connected with husbands’ FertiQoL (*β* = 0.150, 95% CI: 0.024 to 0.277, *p* = 0.021), whereas wives’ VPA maintained a significant inverse partner relationship (*β* = −0.184, 95% CI: −0.307 to −0.061, *p* = 0.004). All other actor and partner links were not statistically significant (*p* > 0.05). Furthermore, as presented in Supplementary Table 4, both models demonstrated acceptable to well-fit characteristics according to standard evaluation criteria. Specifically, Model 2 showed an improved overall fit compared to Model 1, with a χ^2^/df of 2.519, an SRMR of 0.047, and an RMSEA of 0.065, all falling well within acceptable thresholds.

**Table 4:** Actor-partner associations of physical activity on FertiQoL scores (*N* = 364 dyads).

Actor-partner associations	Model 1^a^			Model 2 ^b^		
	Unstandardized *B* (SE)	Standardized *β* (95% CI)	*p*	Unstandardized *B* (SE)	Standardized *β* (95% CI)	*p*
Actor’s associations						
Husbands’ LPA → Husbands’ FertiQoL	0.371 (0.122)	0.182 (0.066, 0.297)	0.002	0.420 (0.137)	0.205 (0.076, 0.335)	0.002
Husbands’ MPA → Husbands’ FertiQoL	−0.006 (0.077)	−0.005 (−0.136, 0.126)	0.941	−0.009 (0.076)	−0.008 (−0.138, 0.122)	0.904
Husbands’ VPA → Husbands’ FertiQoL	−0.016 (0.041)	−0.025 (−0.151, 0.101)	0.699	−0.018 (0.042)	−0.028 (−0.158, 0.102)	0.672
Wives’ LPA → Wives’ FertiQoL	0.080 (0.113)	0.044 (−0.077, 0.164)	0.478	0.179 (0.123)	0.098 (−0.034, 0.229)	0.147
Wives’ MPA → Wives’ FertiQoL	0.116 (0.093)	0.084 (−0.047, 0.214)	0.209	0.103 (0.093)	0.074 (−0.057, 0.205)	0.267
Wives’ VPA → Wives’ FertiQoL	−0.020 (0.057)	−0.024 (−0.151, 0.104)	0.719	−0.027 (0.057)	−0.032 (−0.160, 0.097)	0.629
Partner’s associations						
Husbands’ LPA → Wives’ FertiQoL	0.186 (0.115)	0.100 (−0.020, 0.220)	0.104	0.245 (0.128)	0.132 (−0.003, 0.266)	0.057
Husbands’ MPA → Wives’ FertiQoL	−0.055 (0.072)	−0.053 (−0.188, 0.083)	0.445	−0.047 (0.071)	−0.046 (−0.180, 0.089)	0.506
Husbands’ VPA → Wives’ FertiQoL	0.002 (0.039)	0.003 (−0.127, 0.133)	0.968	−0.007 (0.040)	−0.011 (−0.145, 0.123)	0.869
Wives’ LPA → Husbands’ FertiQoL	−0.146 (0.120)	−0.073 (−0.189, 0.044)	0.223	−0.140 (0.131)	−0.069 (−0.197, 0.058)	0.288
Wives’ MPA → Husbands’ FertiQoL	0.226 (0.099)	0.148 (0.022, 0.273)	0.022	0.230 (0.099)	0.150 (0.024, 0.277)	0.021
Wives’ VPA → Husbands’ FertiQoL	−0.180 (0.060)	−0.189 (−0.311, −0.066)	0.003	−0.176 (0.061)	−0.184 (−0.307, −0.061)	0.004

^a^Model 1 was an unadjusted model; ^b^Model 2 was adjusted for age, BMI, education, annual income, marital status.

*B*, unstandardized path coefficient; SE, Standard error; *β*, standardized path coefficient; CI, Confidence interval; FertiQoL, fertility-related quality of life; LPA, light physical activity; MPA, moderate physical activity; VPA, vigorous physical activitivity.

## 4 Discussion

To our knowledge, this is one of the first studies to employ the APIM to explore the dyadic relationship between physical activity and fertility quality of life among infertile couples. Our findings revealed that females generally exhibited lower levels of both physical activity and FertiQoL compared to their male partners, yet significant positive correlations existed within couples for both metrics. Notably, physical activity intensities were significantly associated with most FertiQoL domains at the individual level, particularly LPA. Crucially, the dyadic analysis identified gender-specific and intensity-dependent partner associations. Specifically, wives’ MPA showed a positive cross-partner connection with husbands’ FertiQoL, whereas their VPA was paradoxically linked to lower FertiQoL in husbands. Overall, these results suggest that physical activity functions not only as an individual lifestyle factor, but as a critical dyadic dynamic shaping inter-spousal FertiQoL during fertility care. By disentangling intensity-specific associations and uncovering these inter-spousal partner effects, our findings advance the field beyond broad individual-level associations, offering actionable evidence for couple-centered lifestyle interventions during ART treatment.

Regarding QoL, our findings are largely consistent with established evidence indicating that infertile couples, particularly women, exhibit significantly lower FertiQoL compared to the general population or their male partners (15, 45, 46). This disparity may stem from prolonged ART interventions, treatment uncertainty, and social stigma associated with infertility, which render women more susceptible to severe impairments in quality of life. Specifically, these vulnerabilities are most pronounced among younger women with lower educational attainment (47, 48), and those with a sustained child wish (49). Previous evidence suggests that the anxiety, stress, and depression accompanying an infertility diagnosis can profoundly influence health behaviors and lifestyle choices within couples (50). Regarding physical activity, the observed disparity between spouses may be attributed to the unique physical and psychological burdens faced by women during preparation for pregnancy and treatment. For instance, fatigue or discomfort arising from physical activity, coupled with a profound fear of fetal harm, represents a significant barrier to maintaining active lifestyles among women seeking to conceive (51). Furthermore, a history of miscarriage, alongside infertility and its related treatment experiences, may further reinforce these cautious behavioral patterns (52–54). Given the rising prevalence of infertility and the distinct barriers faced by women, addressing suboptimal physical activity and psychological distress through targeted interventions remains a critical priority for future reproductive health research.

While the positive link between physical activity and quality of life is well-established across general adult populations (55–58), the present study extends this paradigm to the uniquely vulnerable context of infertile couples. Regarding the individual-level findings, our findings reveal that the association between physical activity and FertiQoL exhibits notable nuances depending on exercise intensity. Accumulating evidence consistently links LPA to superior overall QoL, physical and social functioning, and subjective well-being, particularly in older and clinical populations (59–61). Similarly, studies on postmenopausal women demonstrate that LPA correlates significantly across all QoL domains, with broader connections than MPA and VPA (62). Although higher-intensity activities often exert stronger effects on specific physical or physiological metrics, they do not necessarily hold a universal advantage in broader psychosocial or life-satisfaction domains. Aligning with this evidence, LPA showed the most extensive connections with various FertiQoL domains in our study, including emotional, mind–body, relational, social, environment, and core FertiQoL, highlighting its prominent role in supporting the well-being of individuals facing fertility challenges. In contrast, MPA and VPA exhibited more selective or domain-specific associations with dimensions such as mind–body, tolerability, or treatment FertiQoL. This observed nuance is further supported by research differentiating the impacts of specific physical activity types. For instance, studies on patients undergoing *in vitro* fertilization (IVF) have shown that occupational, transportation, and household physical activity are specifically associated with reduced levels of depression, anxiety, and interpersonal sensitivity (63). Similarly, studies on women in their second and third trimesters found that while VPA correlates with psychological and social QoL, occupational physical activity is specifically associated with superior scores in the physical QoL (64). A study in Hungary among women with infertility demonstrated that recreational physical activity was weakly yet significantly and positively associated with all QoL dimensions (29). Overall, these findings underscore the therapeutic value of integrating diverse physical activities into the daily lives of infertile couples and highlight the importance of tailored exercise guidelines, suggesting that even modest domain-specific score gains in FertiQoL may support real-world psychological adaptation during ART treatment.

Our findings identified gender-specific and intensity-dependent actor-partner patterns, demonstrating that physical activity is intricately linked to FertiQoL within infertile couples. While previous literature primarily applied the APIM to internal traits such as resilience (65, 66) or infertility-related stress and dyadic coping (32), our study extends this paradigm to behavioral domains. Specifically, higher LPA was positively connected with husbands’ own FertiQoL, representing a notable actor effect and highlighting the individual benefits of lower-intensity engagement within marital dyads facing infertility. More strikingly, our model revealed contrasting partner effects exerted by wives on their husbands’ QoL. While wives’ MPA showed a positive partner association with husbands’ FertiQoL, wives’ VPA maintained a significant inverse partner relationship. Grounded in the Spillover-Crossover Model (SCM) and APIM, this divergence may be explained by the differing social and psychological implications of exercise intensity within a relationship (67, 68). Compared to VPA, MPA is often more seamlessly integrated into the couple’s daily life and is associated with enhanced partner support, joint participation, and positive relational experiences, thereby fostering a more supportive dyadic environment (69–71). This reinforces the broader psychological consensus that partners in fertility struggles do not cope in isolation; rather, physical and behavioral patterns are mutually shared and transmitted across the marital dyad, thereby validating our observed partner associations (32, 65, 66). While moderate exercises may align with shared daily routines, the unexpected inverse association involving wives’ VPA warrants cautious interpretation. It is plausible to hypothesize that high-intensity activity by the female partner might reflect underlying fatigue, increased health-related anxiety, or differing behavioral synchronization within couples facing fertility challenges. Because our cross-sectional design precludes causal inference, several alternative explanations must be acknowledged, such as residual confounding, reverse causation, or unmeasured treatment-stage differences. Therefore, these findings should be regarded as preliminary. Future longitudinal or intervention studies are needed to test these potential mechanisms rather than assuming direct causation. Ultimately, these findings highlight the necessity of a shift from individual-focused advice to dyadic-centered counseling that emphasizes synchronized communication and intensity to optimize FertiQoL for both partners.

The present study has several notable strengths. Primarily, it provides a comprehensive assessment of physical activity by distinguishing between different intensities, rather than treating exercise as a homogenous variable. Furthermore, by adopting a distinguishable dyadic perspective and utilizing the APIM framework, this study treats infertile couples as a single unit, thereby capturing complex interpersonal dynamics that individual level analyses often overlook. Despite these strengths, several limitations warrant consideration. Firstly, the cross-sectional design precludes the determination of causal relationships between physical activity and FertiQoL. Future prospective or longitudinal studies are essential to validate these associations and establish true causality. Secondly, this was a single-center study conducted within a specific clinical setting, which may introduce selection bias and limit the generalizability of the findings to broader populations. Multicenter studies with more diverse and representative cohorts are needed to replicate and bolster our results. Thirdly, physical activity and FertiQoL were assessed using self-report measures, which are inherently prone to recall and social desirability biases. Although rigorous quality control was implemented throughout data collection, future research should consider incorporating objective measures, such as accelerometers, to provide a more precise and unbiased quantification of physical activity levels. Fourthly, while statistically significant associations were identified between physical activity levels and FertiQoL scores, an established Minimal Clinically Important Difference (MCID) for FertiQoL is currently lacking. Consequently, whether these score variations correspond to clinically meaningful changes in long-term psychological outcomes warrants further validation through prospective clinical studies. Finally, the possibility of residual confounding from unmeasured clinical factors and other uncaptured psychosocial dynamics cannot be completely ruled out. Future investigations should adjust for a more comprehensive range of clinical and psychosocial covariates to further clarify these complex dyadic associations.

## 5 Conclusion

In conclusion, this study underscores the significant observational associations between various intensities of physical activity and FertiQoL among infertile couples. While individual-level physical activity is broadly beneficial, the dyadic analysis reveals that wives’ MPA and VPA exert contrasting partner effects on their husbands’ FertiQoL. Given the cross-sectional design, these findings do not establish direct causality; however, they highlight the potential value of incorporating a dyadic perspective when evaluating lifestyle factors in infertility care. Future prospective studies and intervention trials are needed to determine whether couple-coordinated physical activity can effectively optimize quality of life for both partners during infertility treatment.

## Data Availability

The raw data supporting the conclusions of this article will be made available by the authors, without undue reservation.
