# Supplementary material for: Dyadic associations of physical activity intensity with fertility-related quality of life in infertile couples: an APIM analysis
Source: Front Public Health. 2026 Aug 24;14:1923237. doi: 10.3389/fpubh.2026.1923237 (PMC13547252; doi:10.3389/fpubh.2026.1923237)
Supplement: Supplementary file 1 [file Table_1.DOCX]

**Table S1.** Metabolic equivalent of task (MET) values assigned to physical activities in the questionnaire.

| Items | MET | PA |
| --- | --- | --- |
| 1-1 | 2.0 | Walking (very slow / mobility-impaired) |
| 1-2 | 2.8 | Walking (slow / slower than average) |
| 1-3 | 3.5 | Walking (normal / average pace) |
| 1-4 | 5.0 | Walking (Brisk / faster than average) |
| 1-5 | 7.0 | Walking (Very fast) |
| 2 | 7.0 | Running |
| 3 | 7.5 | Bicycling |
| 4 | 4.9 | Racquet and team sports (e.g., basketball, soccer, tennis, and table tennis) |
| 5 | 6.0 | Swimming |
| 6 | 6.0 | Aerobics or High intensity interval training. |
| 7 | 2.9 | Traditional and mind-body exercises (Tai Chi, yoga, and square dancing, etc.) |
| 8 | 3.5 | Domestic activities (e.g., cooking and cleaning) |
| 9 | 8.0 | Heavy carrying |
| 10 | 5.0 | Resistance training (e.g., dumbbells, squats, and weighted leg exercises) |
| 11 | 1.4 | Watching TV, mobile phone, or tablet |
| 12 | 1.5 | Desk work |
| 13 | 1.4 | Computer use |

Abbreviations: MET, metabolic equivalent of task; PA, physical activity.

**Table S2.** Spearman rank correlations of physical activity intensities between partners in infertile couples (*N* = 364 dyads).

| Variables | Wives’ LPA | Wives’ MPA | Wives’ VPA | Wives’ MVPA | Wives’ TPA |
| --- | --- | --- | --- | --- | --- |
| Husbands’ LPA | 0.362^⁎⁎^ | 0.112^⁎^ | -0.004 | 0.083 | 0.235^⁎⁎^ |
| Husbands’ MPA | 0.208^⁎⁎^ | 0.166^⁎⁎^ | 0.095 | 0.155^⁎⁎^ | 0.201^⁎⁎^ |
| Husbands’ VPA | -0.006 | 0.057 | 0.124^⁎^ | 0.084 | 0.056 |
| Husbands’ MVPA | 0.131^⁎^ | 0.133^⁎^ | 0.107^⁎^ | 0.134^⁎^ | 0.153^⁎⁎^ |
| Husbands’ TPA | 0.227^⁎⁎^ | 0.158^⁎⁎^ | 0.090 | 0.148^⁎⁎^ | 0.210^⁎⁎^ |

Note: Data are presented as Spearman rank correlation coefficients (*r*_s_). *p* < 0.05 and *p* < 0.01 are indicated by * and **, respectively.

Abbreviations: LPA, light physical activity; MPA, moderate physical activity; VPA, vigorous physical activity; MVPA, moderate-to-vigorous physical activity; TPA, total physical activity.

**Table S3.** Pearson correlation coefficients of fertility-related quality of life scores between partners in infertile couples (*N* = 364 dyads).

| Variables | Wives’ Emotional | Wives’  Mind-body | Wives’ Relational | Wives’  Social | Wives’ Environment | Wives’ Tolerability | Wives’  Core FertiQoL | Wives’  Treatment FertiQoL | Wives’  Total FertiQoL |
| --- | --- | --- | --- | --- | --- | --- | --- | --- | --- |
| Husbands’ Emotional | 0.198**^⁎⁎^** | 0.197**^⁎⁎^** | 0.233**^⁎⁎^** | 0.273**^⁎⁎^** | 0.233**^⁎⁎^** | 0.165**^⁎⁎^** | 0.266**^⁎⁎^** | 0.247**^⁎⁎^** | 0.279**^⁎⁎^** |
| Husbands’ Mind-body | 0.203**^⁎⁎^** | 0.253**^⁎⁎^** | 0.162**^⁎⁎^** | 0.264**^⁎⁎^** | 0.185**^⁎⁎^** | 0.181**^⁎⁎^** | 0.269**^⁎⁎^** | 0.225**^⁎⁎^** | 0.275**^⁎⁎^** |
| Husbands’ Relational | 0.193**^⁎⁎^** | 0.152**^⁎⁎^** | 0.280**^⁎⁎^** | 0.271**^⁎⁎^** | 0.179**^⁎⁎^** | 0.093 | 0.257**^⁎⁎^** | 0.169**^⁎⁎^** | 0.249**^⁎⁎^** |
| Husbands’ Social | 0.236**^⁎⁎^** | 0.241**^⁎⁎^** | 0.248**^⁎⁎^** | 0.351**^⁎⁎^** | 0.235**^⁎⁎^** | 0.183**^⁎⁎^** | 0.320**^⁎⁎^** | 0.258**^⁎⁎^** | 0.324**^⁎⁎^** |
| Husbands’ Environment | 0.144**^⁎⁎^** | 0.124**^⁎^** | 0.221**^⁎⁎^** | 0.189**^⁎⁎^** | 0.300**^⁎⁎^** | 0.128**^⁎^** | 0.196**^⁎⁎^** | 0.266**^⁎⁎^** | 0.230**^⁎⁎^** |
| Husbands’ Tolerability | 0.090 | 0.145**^⁎⁎^** | 0.048 | 0.138**^⁎⁎^** | 0.186**^⁎⁎^** | 0.166**^⁎⁎^** | 0.131**^⁎⁎^** | 0.215**^⁎⁎^** | 0.164**^⁎⁎^** |
| Husbands’ Core FertiQoL | 0.241**^⁎⁎^** | 0.250**^⁎⁎^** | 0.259**^⁎⁎^** | 0.335**^⁎⁎^** | 0.242**^⁎⁎^** | 0.187**^⁎⁎^** | 0.323**^⁎⁎^** | 0.265**^⁎⁎^** | 0.328**^⁎⁎^** |
| Husbands’ Treatment FertiQoL | 0.147**^⁎⁎^** | 0.168**^⁎⁎^** | 0.171**^⁎⁎^** | 0.206**^⁎⁎^** | 0.306**^⁎⁎^** | 0.183**^⁎⁎^** | 0.206**^⁎⁎^** | 0.301**^⁎⁎^** | 0.247**^⁎⁎^** |
| Husbands’ Total FertiQoL | 0.234**^⁎⁎^** | 0.248**^⁎⁎^** | 0.256**^⁎⁎^** | 0.327**^⁎⁎^** | 0.282**^⁎⁎^** | 0.202**^⁎⁎^** | 0.317**^⁎⁎^** | 0.299**^⁎⁎^** | 0.333**^⁎⁎^** |

Note: Data are presented as Pearson correlation coefficients (*r*). *p* < 0.05, *p* < 0.01, and *p* < 0.001 are indicated by *, **, and ***, respectively.

Abbreviations: FertiQoL, fertility-related quality of life.

**Table S4.** Model fit indices for the structural equation models (*N* = 364 dyads)

| Fit coefficients | λ^2^/df | SRMR | RMSEA | GFI | AGFI | TLI | CFI | IFI | NFI |
| --- | --- | --- | --- | --- | --- | --- | --- | --- | --- |
| Reasonable fit | <5.000 | <0.100 | <0.100 | >0.800 | >0.800 | >0.800 | >0.800 | >0.800 | >0.800 |
| Well fit | <3.000 | <0.080 | <0.080 | >0.900 | >0.900 | >0.900 | >0.900 | >0.900 | >0.900 |
| Model 1 | 3.471 | 0.058 | 0.082 | 0.904 | 0.813 | 0.872 | 0.895 | 0.896 | 0.860 |
| Model 2 | 2.519 | 0.047 | 0.065 | 0.930 | 0.800 | 0.856 | 0.881 | 0.883 | 0.820 |

Note: Model 1 was an unadjusted model including only the primary predictor and outcome variables within the APIM framework. Model 2 was adjusted for potential confounders, including age, BMI, educational level, annual household income, and marital status. Abbreviations: λ^2^/df, chi-square/degrees of freedom; SRMR, standardized root mean square residual; RMSEA, root-mean-square error of approximation; GFI, goodness of fit index; AGFI, adjusted goodness of fit index; TLI, Tucker-Lewis index; CFI, comparative fit index; IFI, incremental fit index; NFI, normed fit index.
